# Students’ Reflection on Online Clinical Scenario Sessions As Complementary to Online Anatomy Practical Sessions During the COVID-19 Pandemic at the University of Khartoum in 2021

**DOI:** 10.7759/cureus.94295

**Published:** 2025-10-10

**Authors:** Haythem M Ahmed, Abdulshakor S Ali, Khalid A Awad, Elimam Elghazali S Mustafa, Kamal Eldeen M Dahab

**Affiliations:** 1 General Surgery, Oxford University Hospitals NHS Foundation Trust, Oxford, GBR; 2 Anatomy, University of Khartoum, Khartoum, SDN; 3 Trauma and Orthopaedics, Oxford University Hospitals NHS Foundation Trust, Oxford, GBR

**Keywords:** anatomy instruction, case-based learning, covid-19, online clinical scenarios, virtual medical education

## Abstract

Introduction

The COVID-19 pandemic necessitated a rapid shift to virtual teaching in medical education. At the Faculty of Medicine, University of Khartoum, all face-to-face sessions were replaced with online instruction during the second wave to reduce transmission risks. This study aimed to evaluate student reflections on the use of online clinical scenarios as a complementary tool to virtual anatomy practical sessions.

Methods

A cross-sectional analytic study was conducted among first-year medical students. Five online clinical scenarios were delivered synchronously via Zoom (Zoom Video Communications, San Jose, CA) to supplement virtual anatomy teaching. At the end of the academic year, students' satisfaction and feedback were gathered using a structured, self-administered questionnaire developed through Google Forms (Google, Inc., Mountain View, CA).

Results

More than half of the respondents (52.7%, n = 68) agreed or strongly agreed that the sessions enhanced their retention of anatomical knowledge and facilitated the study of gross anatomy, while 17.1% (n = 22) disagreed or strongly disagreed (p = 0.003; 95% CI: 0.45-0.60). The perceived effectiveness of the online clinical scenarios was comparable to traditional face-to-face sessions, with nearly two-thirds of participants (64.3%, n = 83) recommending their continued use (p = 0.001; 95% CI: 0.58-0.70).

Conclusion

Students responded positively to the incorporation of online clinical scenarios, viewing them as effective and comparable to conventional teaching methods. These findings support the integration of case-based learning into virtual anatomy education during and beyond pandemic-related disruptions.

## Introduction

The introduction of case-based learning or clinical scenarios station within the practical gross anatomy sessions at the University of Khartoum was first done in 2018, following a study done by Awad and Saeed [1] that showed a high level of students’ satisfaction. The clinical scenarios were designed to stimulate critical thinking, and they were related to the topic studied in the session [1].

On January 30, 2020, COVID-19 was declared to be a global health emergency by the World Health Organization [2]. This novel coronavirus was first noticed in Wuhan, China, in late December 2019 [3]. Despite the strict public health measures that have been taken by governments to contain the pandemic, including quarantine, testing, and social distancing, the worldwide incidence of COVID-19 has increased rapidly, with a global death toll of 2,060,731 as of January 20, 2021 [4].

Since the beginning of its first wave, the widely spreading COVID-19 pandemic has markedly affected medical education all over the world. One of the main public health measures that has been taken to reduce its spread was the country-wide lockdown that, in turn, necessitated the closure of university campuses with their medical schools and departments, with shifting all the educational activities into online sessions [5].

Due to its dilapidated health system and poor living conditions, Sudan was one of the countries that has been markedly affected during this pandemic, with a total number of confirmed cases reaching 25,730 and total deaths of 1,576 as of December 31, 2020 [6].

As a result of this pandemic, medical schools were compelled to apply a rapid transition to virtual teaching methods [5]. This represented a real challenge to medical schools, which in turn were working very hard to deliver high-quality education to students virtually [7]. The Faculty of Medicine University of Khartoum was one of those medical schools, and since October 2020, the anatomy department staff members were doing their best to provide a useful, safe, and reliable alternative method for teaching during the current circumstances.

A systematic review on the application and effectiveness of virtual teaching during the COVID-19 pandemic was done by Wilcha [8]. A total of 39 studies were included in this review based on certain inclusion criteria. From the available evidence, he concluded that virtual teaching is an effective alternative during the period of COVID-19, and institutions are working to further develop these resources to improve student engagement and interaction. Wilcha said that medical schools should take on a more holistic approach to student education, and the psychological impact of COVID-19 on students’ mentality should be considered [8].

A study that was done by Khalil et al. [7] at Qassim University in Saudi Arabia to explore medical students’ perceptions regarding the effectiveness of synchronized online sessions at Unaizah College of Medicine and Medical Sciences found that synchronized online classes were well-accepted by the medical students. The students agreed that online sessions helped them in managing their time with an apparent impact on their performance; however, they indicated that some challenges were encountered, including methodological, content perception, technical, and behavioral challenges during sessions and online exams [7]. A similar study at the same university, which was done by Elzainy et al. [9], demonstrated a marked increase in the mean PBL grades for female students during the online sessions. Most of the basic year students and staff members expressed a high level of satisfaction with virtual teaching and assessment methods used [9].

Verma et al. conducted another research in India exploring students’ perception regarding online teaching adopted during the COVID-19 pandemic [10]. Almost all the participants agreed that the online sessions were relevant and suited their educational needs. Most of them found that the online sessions were time-saving. Disadvantages of online sessions were mostly found to be related to the technical issues, teachers’ modest knowledge about the technology, lack of interactive teaching, and easy distraction [10].

Another Indian study that was conducted to evaluate the challenges encountered by the first-year medical and dental students during their virtual anatomy classes demonstrated that most of the participants expressed their longing for the traditional anatomy teaching methods (dissection courses, face-to-face lectures, and interaction with mentors). The students faced some difficulty in the topics completed without dissections, models, microscopic slides, and other modalities. In addition, the majority of the students struggled with time management and felt a lack of confidence and self-motivation [11].

Lima et al. conducted a study in Brazil using synchronous and asynchronous activities through different online tools to help the students understand the Human Physiology course [12]. The study used Zoom (Zoom Video Communications, San Jose, CA), Mentimeter (Mentimeter AB, Stockholm, Sweden), LT Platform (ADInstruments, Dunedin, New Zealand), Socrative Student (Showbie Inc., Edmonton, Canada), YouTube (Google LLC, Mountain View, CA), Facebook/Instagram (Meta Platforms Inc., Menlo Park, CA), and Lucidchart (Lucid Software Inc., South Jordan, UT) as online tools. Among the used tools, the students selected the LT Platform, Zoom, Mentimeter, and YouTube as the best online tools to be used in online physiology teaching [12].

## Materials and methods

This was a cross-sectional analytic study that was carried out among the first year (semester 2) medical students at the Faculty of Medicine University of Khartoum (batch 96). The total number of first-year students is 325, among them 102 are males (31.4%) and 223 are females (68.6%).

The Faculty of Medicine University of Khartoum runs a six-year undergraduate academic program, and each academic year consists of two semesters. Basic biomedical sciences (anatomy, physiology, and biochemistry) are given during the first two academic years, particularly in semesters 2, 3, and 4. In addition to gross anatomy, the Anatomy Department at the Faculty of Medicine University of Khartoum is responsible for teaching developmental anatomy (embryology), histology, and radiological anatomy for the first and second year medical students.

The gross anatomy practical sessions at the Faculty of Medicine University of Khartoum are used to be student-centered. Each anatomy practical session is composed of five stations, four of which are prosection-based stations and the fifth station is the newly introduced clinical scenario station [1].

Due to the first wave of the COVID-19 pandemic, the Sudanese government had announced a country-wide lockdown effective from March 15, 2020; this decision involved suspending all face-to-face teaching activities in all Sudanese universities. The decision to start online teaching in the Sudanese universities has been delayed for many reasons, including a lack of essential infrastructure in most of the universities, poor network connectivity in Sudan, high cost of network subscription, and lack of network accessibility among some of the students due to its high cost. The decision to start online teaching was made in October 2020 after solving some of the abovementioned reasons.

The Anatomy Department at the University of Khartoum created an educational channel on Telegram (Pavel Durov and Nikolai Durov, Dubai, UAE), YouTube (Google Inc., Mountain View, CA), and Facebook (Meta Platforms, Inc., Menlo Park, CA). Anatomy lectures were given in the form of recorded video format or recorded PowerPoint presentations (Microsoft® Corp., Redmond, WA) that contain voice records. They were uploaded to the batch 96 educational Telegram channel. The four prosection-based stations in the anatomy practical sessions were given asynchronously through demonstrative videos that have been recorded in the dissection room of the University of Khartoum by the Anatomy Department staff members. Those videos were uploaded to the official anatomy department channel on YouTube and delivered to the students through Moodle (Moodle Pty Ltd, Perth, Australia).

To augment those online anatomy practical sessions, online clinical scenarios were given synchronously through Zoom meetings. Five online sessions were given in the period from October 21 to December 27, 2020, with a period of two weeks between sessions. The sessions were prepared and given by the Anatomy Department staff members. All those sessions were recorded and uploaded to the batch 96 educational Telegram channel to be available for those who could not attend them synchronously. Each session was approximately one hour in length, with the session’s mean duration being 68 minutes. The scenarios were designed to stimulate critical thinking and to integrate anatomical facts with clinical practice. The sessions were in the form of case-based discussion between the tutors and the students, and whenever possible, scenarios were supported by relevant imaging studies (e.g., X-rays, CT scans). Students’ satisfaction and reflections on the online clinical scenarios were assessed at the end of the academic year.

Data were collected using a self-administered structured questionnaire. The questionnaire was openly accessible online and did not require permission for its use, consisting of four parts: (1) Demographic data: Students were asked to enter their age, gender, describe their IT skills, and state whether they had previously participated in any online courses. (2) Advantages and disadvantages of e-learning: Students were given six sets of options regarding the advantages and disadvantages of e-learning, from which they could choose as many as were true for them. (3) Benefits gained from the online clinical scenarios: using five-point Likert scale (where 1 = strongly disagree, 5 = strongly agree), the questions were designed to cover all aspects of learning of gross anatomy as intended learning outcomes achievement, students’ satisfaction, enjoyment, relevance to the practical session topic, stimulation of deep learning and relation to future practice. (4) Comparison between the effectiveness of face-to-face clinical scenarios (station 5 in the DR practical session) and that of the online clinical scenarios’ sessions using a five-point Likert scale (where 1 = extremely ineffective, 5 = extremely effective).

The questionnaire was available to all the first-year medical students in their Telegram group, which was made by the Anatomy Department of the Faculty of Medicine University of Khartoum for virtual teaching purposes. All the students were asked to fill in the questionnaire. The number of students who agreed to participate in this study and filled in the questionnaire was 129.

Data was managed and analyzed using SPSS version 26 (IBM Corp., Armonk, NY). Analysis included descriptive statistics and a t-test that were used to compare the effectiveness of online and face-to-face clinical scenarios. p < 0.05 was considered statistically significant.

## Results

The total number of participants who responded to the online questionnaire was 129 students (83 females, 42 males, four preferred not to specify their gender) out of 325 (response rate = 39.7%). The mean age of the participants was 19.23 years ± 0.74 years.

Most of the participants (58 participants = 45%) described their IT skills as average, 11 of them (8.5%) described it as bad, and three of them (2.3%) described it as very bad, whereas 40 of them (31%) described it as good, 17 of them (13.2%) described it as very good. A total of 55 of the participants (42.6%) have previously participated in e-learning before the pandemic, whereas 74 of them (57.4%) have no previous experience with virtual learning.

The advantages of e-learning from the students’ perspective were the ability to stay at home (79.8%, n = 103), ability to record a meeting or session (67.4%, n = 87), continuous access to online materials (66.7%, n = 86), the opportunity to learn at your own pace (62 %, n = 80), and comfortable surroundings (52.7%, n = 68). On the other hand, most of the participants chose poor network connectivity in our country (71.3%, n = 92), lack of self-discipline (51.9%, n = 67), reduced interaction with teachers (44.2%, n = 57), and technical issues (40.3%, n = 52) as the main disadvantages. Table 1 and Table 2 summarize the advantages and disadvantages of e-learning from the students’ perspective, respectively. 

**Table 1:** Advantages of e-learning

Advantages of e-learning		
Ability to stay at home	79.8%	103
Ability to record a meeting or session	67.4%	87
Continuous access to online materials	66.7%	86
Opportunity to learn at your own pace	62%	80
Comfortable surroundings	52.7%	68
Reduced money, time, physical and emotional effort	0.8 %	1
Having enough time for discussion with the tutor during synchronous sessions	0.8%	1
Feeling confident in answering and participation	0.8%	1
Less distraction	0.8%	1
Avoiding social interactions	0.8%	1

**Table 2:** Disadvantages of e-learning

Disadvantages of e-learning		
Poor network connectivity in our country	71.3 %	92
Lack of self-discipline	51.9 %	67
Reduced interaction with teachers	44.2 %	57
Technical issues	40.3 %	52
Social isolation	36.4 %	47
Poor learning conditions at home	32.6 %	42
Network subscription costs	0.8%	1
Time management	0.8%	1

Most of the students’ agreements regarding benefits gained from the online clinical scenarios were to the item “link anatomy to clinical practice,” followed by “online clinical scenarios should be continued.” While the fewest agreements were to the item “achievement of intended learning outcomes.” Students’ agreements ranged from 69% (n = 89) to 39.5% (n = 51), while disagreements ranged from 27.2% (n = 35) to 11.6%. (n = 15). Table 3 summarizes students’ responses to the benefits gained from the online clinical scenarios: using five-point Likert scale questions.

**Table 3:** Responses of the study participants to different items of Likert scale questions

Item	Strongly agree	agree	Can’t decide	disagree	Strongly disagree
Link anatomy to clinical practice	26.4	42.6	19.4	6.2	5.4
Online clinical scenarios should be continued	36.4	27.9	13.2	12.4	10.1
Online clinical scenarios are enjoyable	38	24.8	12.4	14.7	10.1
Increased understanding of gross anatomy	20.2	41.1	24.8	8.5	5.4
Increased satisfaction about gross anatomy	13.2	42.6	26.4	10.1	7.8
Increased interest in anatomy	19.4	34.1	25.6	11.6	9.3
Stimulation of deep knowledge	20.2	32.6	27	13.2	7
Help in retention of anatomical facts	16.3	36.4	30.2	10.9	6.2
The scenarios were at their level and they were able to link them to the dissection room sessions	15.5	32.6	24.8	19.4	7.8
Helped in achieving intended learning outcomes	9.3	30.2	36.4	15.5	8.5

Upon asking the participants to rate the effectiveness of the online versus the offline clinical scenarios session, 63.5% (n = 82) of the participants rated the online clinical scenarios as effective, compared to 55.1% (n = 71) who rated the offline sessions as effective. On the other hand, 17.1% (n = 22) of the participants rated online clinical scenarios as ineffective compared to 17.8% (n = 23) who rated the offline clinical scenarios as ineffective. Upon statistical testing, there was no statistically significant difference between them, and it was clear that the online clinical scenario sessions (M = 3.69) were as effective as the face-to-face sessions (M = 3.56), p = 0.402 (Table 4).

**Table 4:** Comparison between the effectiveness of the online clinical scenarios and face-to-face clinical scenario sessions The t-test is used to compare means.

Teaching method				p-value
Online clinical scenarios		Face-to-face clinical scenarios		
Mean	Standard deviation	Mean	Standard deviation	0.402
3.69	1.082	3.56	1.117	

## Discussion

This cross-sectional study explored first-year medical students’ perceptions of the transition from traditional face-to-face teaching to online clinical scenario sessions during the COVID-19 pandemic at the University of Khartoum.

The demographic distribution of participants reflected the broader student cohort, lending credibility to the generalizability of the findings. Students identified several key advantages of virtual learning, including the ability to maintain social distancing, record sessions for later review, and study at their own pace.

These benefits align with the public health goals of the lockdown period, which aimed to minimize gatherings and reduce transmission risk. Similar findings were reported by Bączek et al. in their study among Polish medical students, where flexibility and accessibility were among the most valued aspects of online education [13]. Khalil et al. also noted that synchronized online learning was well received by Saudi medical students, who appreciated its time efficiency and adaptability despite facing behavioral and methodological challenges [7].

However, the transition was not without its drawbacks. In our Sudanese context, persistent network connectivity issues emerged as the most significant barrier to effective online learning. This was compounded by students’ limited prior exposure to virtual platforms and generally moderate to poor IT skills, which likely contributed to the frequency of technical difficulties. These challenges mirror those reported by Jahan et al. in Oman, where students similarly struggled with technological readiness and infrastructure limitations [14].

Reduced interaction with instructors and peers was another commonly cited disadvantage, reflecting a broader concern about the loss of collaborative learning in virtual environments. Verma et al. highlighted similar issues in their study among Indian medical students, emphasizing the need for more interactive and engaging online formats to maintain student motivation and participation [10]. Lima et al. further supported this by demonstrating that the use of interactive tools such as Zoom, Mentimeter, and YouTube significantly enhanced student engagement and deep learning during quarantine [12].

Interestingly, although most students had never participated in virtual learning prior to the pandemic, their reflections on the online clinical scenario sessions were largely positive. Many reported increased interest in gross anatomy and appreciated the clinical relevance of the scenarios. These findings suggest that, when well-structured, online clinical teaching can effectively complement traditional anatomy education. Elzainy et al. observed similar outcomes at Qassim University, where students responded positively to online assessments and teaching, noting improvements in performance and satisfaction [9].

Despite these benefits, our study revealed a decline in overall satisfaction compared to the 2018 study by Awad et al., which evaluated face-to-face clinical scenario stations among second-year students [1]. This decline may be attributed to the limitations of virtual learning, particularly reduced interaction and technical challenges. Addressing these issues is essential to improving student engagement and satisfaction. Strategies may include blended learning models, improved digital infrastructure, and targeted training to enhance IT literacy.

Finally, the effectiveness of online clinical scenarios was rated as comparable to traditional face-to-face sessions in terms of knowledge acquisition. This supports the notion that virtual teaching can serve as a viable alternative during disruptions to in-person education, such as pandemics, political instability, or natural disasters. However, it is important to note that while online methods may support cognitive learning, they may fall short in developing hands-on clinical skills and interpersonal competencies, a limitation also noted by Bączek et al. [13].

Limitations

Despite the benefits of online teaching, several significant challenges continue to impact its effectiveness and student satisfaction. These include persistent network connectivity issues, recurring technical difficulties, and the psychological effects of the COVID-19 pandemic. Such obstacles can hinder engagement, disrupt learning continuity, and reduce the overall quality of the online educational experience. Addressing these limitations is essential for improving outcomes and ensuring a more supportive and accessible virtual learning environment.

## Conclusions

Online clinical scenario sessions were well perceived and accepted by the students. As demonstrated by statistical analysis, these integrated sessions are at least as effective as face-to-face teaching in achieving intended learning outcomes, enhancing knowledge acquisition, stimulating deep learning, and improving anatomical understanding and retention. They offer a safe, useful, and reliable alternative to traditional methods, particularly during pandemic-related disruptions.

However, the effectiveness of online teaching is not without challenges. Persistent network connectivity issues, recurring technical difficulties, and the psychological impact of the COVID-19 pandemic can hinder student engagement and disrupt learning continuity. These limitations must be acknowledged and addressed to optimize the virtual learning experience. Future efforts should focus on improving digital infrastructure, providing technical support, and incorporating strategies to support student well-being in remote education environments.
